# Effects of no-tillage sowing on soil properties and forage wheat and Italian ryegrass yields in winter fallow paddy fields

**DOI:** 10.7717/peerj.10573

**Published:** 2021-01-28

**Authors:** Liuxing Xu, Guojian Tang, Jing Tian, Xiaoya Wang, Jianguo Zhang

**Affiliations:** South China Agricultural University, South Pratacultural Research CenterSouth Pratacultural Research Center, South China Agricultural University, Guangzhou, Guangdong, China

**Keywords:** Italian ryegrass, No-tillage, Soil property, Forage wheat, Yield

## Abstract

In South China, it is common practice for the late rice (*Oryza sativa*) that is planted during the summer in the paddy fields after harvest to be used for fallowing or to plant winter forage crops. The land is ploughed before early rice planting. Both forage wheat (*Triticum aestivum*) and Italian ryegrass (*Lolium multiflorum*) have relatively high nutritional value, and planting them in winter fallow paddy fields could potentially address food shortages and provide quality forage for livestock. In this study, we examined the effects of no-tillage sowing 5 days before rice harvest (NB5), no-tillage sowing 1 day after rice harvest (NA1), and conventional tillage sowing (CK) 1 day after rice harvest on forage wheat and Italian ryegrass soil properties, dry matter (DM), and crude protein (CP) yields. Soil and plant samples were collected after three months of crop growth. The results showed that the NB5 and NA1 soil bulk density (0-20 cm soil layer) tended to increase when compared to that of the CK field. The NA1 treatment increased the total soil nitrogen and organic matter content. The enzyme activities and total soil porosity in the no-tillage forage wheat and Italian ryegrass fields tended to decrease, while the no-tillage water content and soil capillary porosity tended to increase when compared to that of the CK field. Overall, planting year significantly influenced soil chemical properties (except for total nitrogen) and enzyme activity, but crop type had no significant effect on soil physical-chemical properties (except for capillary moisture capacity) and enzyme activity. Sowing methods had no significant effects on the crop DM and CP yields. The DM yield was affected by the interaction between planting year and sowing methods, or between sowing methods and crop type. No-tillage also increased the number of species and aboveground weed biomass. We concluded that the best sowing method for forage wheat and Italian ryegrass in winter fallow paddy fields was no-tillage sowing following rice harvest.

## Introduction

The demand for animal proteins in China has rapidly grown with improved living standards and changing food consumption structures. However, animal husbandry developments are usually restricted by shortages in forage supply (Liu et al., 2012). Wheat (*Triticum aestivum*) and Italian ryegrass (*Lolium multiflorum*) are temperate crops that can grow well in the winter fallowed paddy fields in China’s subtropical zones (Xie, 2013). Both the short period between late rice harvest and early rice planting and low winter temperatures limit the growth of food crops, but forage wheat and Italian ryegrass in fallowed paddy fields can be utilized to meet the forage shortage of livestock consumption. Although wheat has been widely used as forage, few studies have focused on its planting techniques. Previous studies on forage wheat mainly concentrated on variety screening (Li, 2015), seeding rate (Li, 2015; Xu, 2016), fertilizer application (Xu, 2016), harvest time (Xie, 2013), and silage utilization (Filya, 2003; Shaani et al., 2017).

The effects of conventional tillage and no-tillage are influenced by factors such as soil and crop type, agricultural systems, crop residues, and climate. Residue retention increases soil organic carbon and total nitrogen concentrations in the upper soil layers (Pu et al., 2019). Alternative subsoiling and no-tillage methods can reduce soil disturbance and improve crop water use efficiency (He et al., 2007), thus increasing crop yields compared to conventional tillage (Qin et al., 2008). Over the last few decades, conventional tillage has negatively affected soil and water environments, causing a loss of soil nutrients, the destruction of soil structure, water pollution, and the decline of microbial diversity in farmland environments (Huang et al., 2010). Furthermore, intensive conventional tillage activities have been connected to reduced land productivity (Manna et al., 2005; Foley et al., 2011). When combining no-tillage sowing with mulch measures or cropping system diversification, the benefits of no-tillage sowing have been demonstrated (Hobbs, Sayre & Gupta, 2008; Nunesa et al., 2018), including its ability to improve soil physical and chemical properties (Zhang et al., 2007), water use efficiency, and crop yield (Hou et al., 2012). However, continuous, annual no-tillage agriculture may ultimately decrease soil quality and crop yield (Ernst et al., 2016). Additionally, conventional tillage and no-tillage crop yields are closely tied to the control of living mulch and weed biomass (Carof et al., 2007). It is worth noting that most previous research results were obtained under intensive double cropping systems, and there is very limited information on the effects of no-tillage sowing on soil and crops under triple cropping systems. When wheat is used for forage, it can be harvested at any time without influencing the early rice planting, but there is very little information available on no-tillage planting techniques for forage wheat in winter fallow paddy fields. Most of the existing literature is focused on growing wheat for grain in arid areas using no-tillage techniques (Lampurlanés et al., 2016; Yang et al., 2020).

In southern China, sowing wheat in winter fallow paddy fields, including rice-wheat double cropping and rice-rice-wheat triple cropping systems (Xu, 2016), has become an important method of land use (Iijima et al., 2005). A proper sowing time has a positive effect on forage yield improvement, and the phenomenon of forage yield decline with a delay in planting time has been often observed (Harper et al., 2017). No-tillage sowing before a rice harvest can effectively prevent late sowing, increase rice growth time, make better use of temperature and light, simplify agronomic measures, and reduce production costs. The soil has more moisture, and sowing wheat before rice harvest positively affects seed germination (Xu et al., 2008).

Although there have been many studies on Italian ryegrass planting techniques (Cinar, Ozkurt & Cetin, 2020), few have looked at no-tillage planting. In addition, the effects of planting forage wheat using no-tillage sowing on soil and crop yield have not been explored. Therefore, in this study, we aimed to compare the effects of different no-tillage methods on soil properties and wheat and Italian ryegrass yields. We hypothesized that in winter fallow paddy fields: (i) short-term, continuous no-tillage sowing would promote better soil health than conventional tillage, and that (ii) no-tillage sowing would have a positive effect on forage wheat and Italian ryegrass yields.

## Materials and Methods

### Experimental site

**Figure 1:**
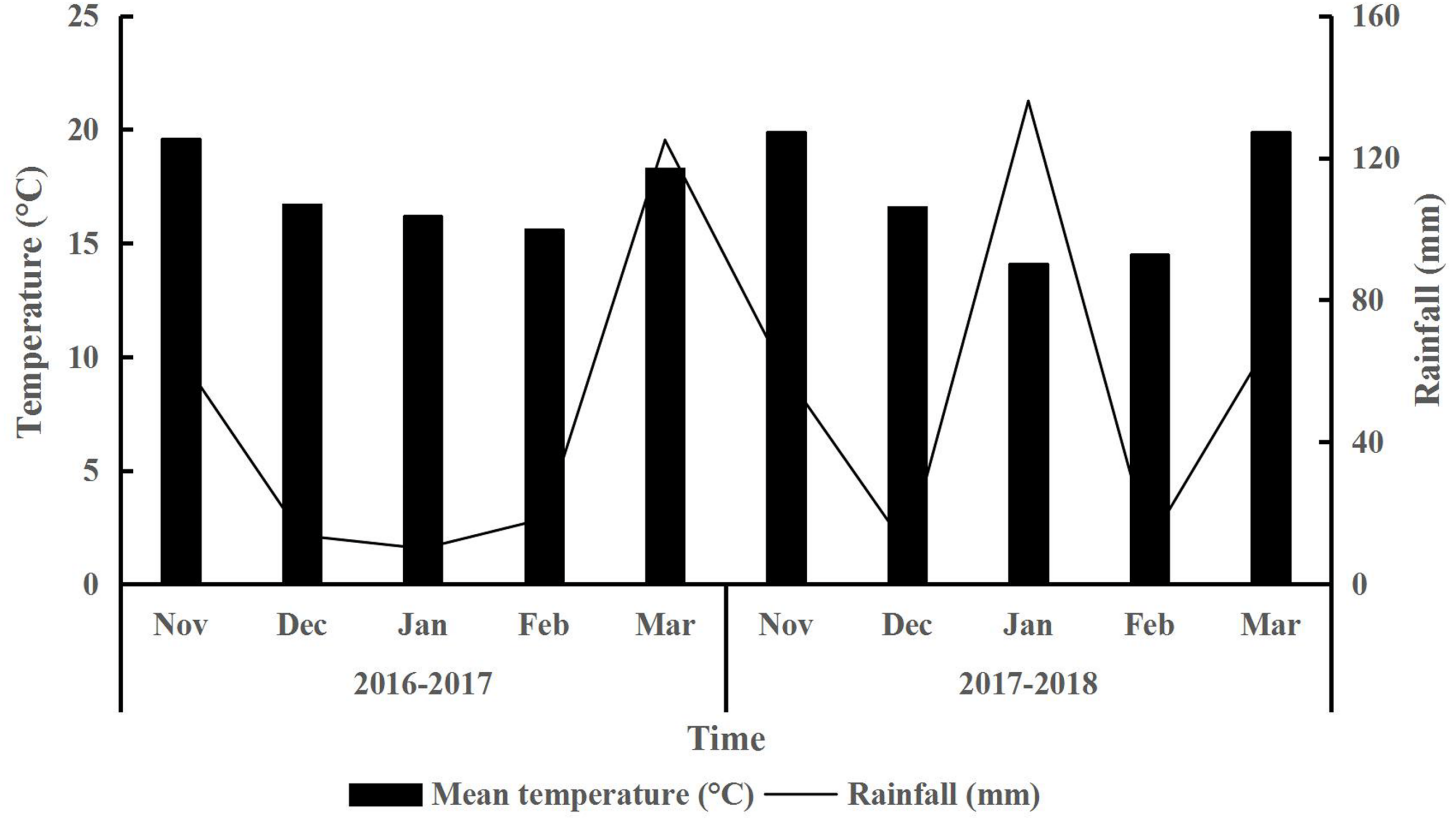
Meteorological data during forage growing period.

The experiment was conducted on an experimental field belonging to South China Agricultural University, Guangzhou, China, located at 23°14′N and 113°38′E. The site was located within a subtropical monsoon, humid climate zone with an annual average temperature of 21.6 °C. The hottest month was July with a monthly average temperature of 29.4 °C, while the coldest month was January with a monthly average temperature of 13.3 °C. The annual average rainfall was 1,967.8 mm and the annual average daylight hours were 1,707.2 h. The site’s soil was classified as paddy soil (Zhang et al., 2014). The site’s soil properties are shown in Table 1. The total rainfall during the forage wheat and Italian ryegrass growing periods (from November to March of the following year) in 2016–2017 and 2017–2018 were 231.1 and 286.2 mm, respectively (Fig. 1). The monthly average temperature during the forage wheat and Italian ryegrass growing periods in 2016–2017 and 2017–2018 were 17.3 and 17.0 °C, respectively. The site’s general cropping practices were to plant early rice in spring (summer harvest) and late rice in summer (autumn harvest), and to either fallow or use the land after the harvest of late rice to plant winter forage crops (to be harvested in spring of the following year). The land was ploughed (20 cm) before the early rice planting.

**Table 1:** Soil properties at the study site in winter fallow fields.

**Measurements**	**2016–2017**	**2017–2018**
pH	5.65 ± 0.10	5.20 ± 0.10
Bulk density (g cm^−3^)	1.48 ± 0.01	1.42 ± 0.05
Organic matter (g kg^−1^)	22.0 ± 0.74	21.1 ± 1.21
Total nitrogen (g kg^−1^)	0.98 ± 0.05	0.92 ± 0.02
Urease (mg g^−1^24 h^−1^)	0.23 ± 0.01	0.30 ± 0.01
Catalase (mg g^−1^20 min^−1^)	0.77 ± 0.30	1.83 ± 0.02
Acid-phosphatase (mg g^−1^24 h^−1^)	54.8 ± 1.17	46.7 ± 0.02
Invertase (mg g^−1^24 h^−1^)	5.22 ± 0.14	6.80 ± 1.75
Bacteria (lg cfu g^−1^ FM)	6.19 ± 0.16	6.11 ± 0.14
Actinomyces (lg cfu g^−1^ FM)	5.35 ± 0.15	5.29 ± 0.11
Fungi (lg cfu g^−1^ FM)	4.50 ± 0.12	4.54 ± 0.07

**Notes.**

FM, fresh matter.

### Wheat and Italian ryegrass planting and management

We used three treatments in this study for forage wheat and Italian ryegrass: (1) forage wheat/Italian ryegrass were sown using the no-tillage method 5 days before rice harvest (NB5); (2) forage wheat/Italian ryegrass were sown using the no-tillage method 1 day after rice harvest (NA1); and (3) forage wheat/Italian ryegrass were sown using a conventional tillage method 1 day after rice harvest (CK). The treatments were arranged in a randomized block design with three replicates. Each plot size was 12 m^2^ (3 × 4 m) in area. The forage wheat (Shimai-1) and Italian ryegrass (Tetraploid) were sown at seeding rates of 285 kg ha^−1^ and 22 kg ha^−1^, respectively. The rice stubble height after harvest was 5 cm. For the CK treatment, we maintained the stubble in the cultivation layer and ploughed the soil (20 cm) with a pneumatic row direct drilling machine equipped with double disc furrow openers. The ground for the CK treatment was ploughed at a depth of approximately 20 cm, while the ground in the no-tillage treatments was not ploughed. We applied compound fertilizer (N: P_2_O_5_: K_2_O = 15:6:8) at rates of 90.0, 36.0, and 48.0 kg ha^−1^ for N, P_2_O_5_, and K_2_O, respectively. Base and jointing fertilizers were applied at a ratio of 6:4. No insecticides and fungicides were used during the experiments. The experiments were conducted for two years. We sowed the NB5 field on November 11, 2016 and November 17, 2017, and the NA1 field on November 16, 2016 and November 22, 2017. We used the same sowing time for the CK field as for the NA1 field. The crops were harvested on March 23, 2017 and March 27, 2018 (Table 2).

**Table 2:** Agricultural operations dates and soil sampling time.

Year	Sowing method	Sowing date	Fertilizing date		Harvesting date	Crop growth time (d)	Time between sowing and the sampling of the soil (d)			
			Base fertilizer	Jointing fertilizer			Enzyme activity and chemical properties	Physical property		
								24-Jan	25-Feb	27-Mar
2016–2017	NB5	11–Nov	24–Dec	22–Feb	21–Mar	133	133	–	–	–
	NA1	16–Nov	24–Dec	22–Feb	21–Mar	128	128	–	–	–
	CK	16–Nov	24–Dec	22–Feb	21–Mar	128	128	–	–	–
2017–2018	NB5	17–Nov	28–Dec	26–Feb	27–Mar	131	131	38	70	100
	NA1	22–Nov	28–Dec	26–Feb	27–Mar	126	126	33	65	95
	CK	22–Nov	28–Dec	26–Feb	27–Mar	126	126	33	65	95

**Notes.**

NB5 notillage sowing on the 5 d before rice harvest NA1 notillage sowing on the 1d after rice harvest CK conventional tillage sowing on the 1d after rice harvest

### Field investigation and sampling

We determined the aboveground forage wheat yield at the dough stage, and the Italian ryegrass on the same day. A 1 m^2^ (1.0 m × 1.0 m) quadrat was randomly selected from each plot to measure the aboveground biomass and weed species richness. We chopped the fresh forage wheat and Italian ryegrass materials to 20 to 30 mm and from each plot, we randomly excavated five soil cores (each 2.5 cm diameter) and mixed them to give a composite sample. After harvesting the forage wheat and Italian ryegrass, we collected the samples to determine soil chemical properties and enzyme activity from the 0–20 cm soil layers during the jointing (January 24, 2018), flowering (February 25, 2018), and dough stages (March 27, 2018). We stored the soil samples set aside for enzyme and microorganism activity analysis at 4 °C, and the other samples were air-dried at room temperature and sieved to 2 mm to determine organic matter and total nitrogen content. The forage wheat and Italian ryegrass were harvested on March 27, 2017 and March 22, 2018, respectively.

### Sample analyses

We dried the forage samples at 70 °C for 48 h in an oven with forced air circulation in order to determine their dry matter (DM) content. Crude protein (CP) and total nitrogen (TN) contents were determined using Kjeldahl nitrogen determination (Nitrogen analyzer KN680, Shandong Jinan Alva Instrument Co., Ltd., Jinan, China; AOAC, 1990). We calculated the DM and CP yields based on the fresh matter yield of the aboveground biomass and the DM and CP contents, respectively. The soil organic matter content was measured using a potassium dichromate heating method (Lu, 2000), and soil water content and bulk density were estimated using fresh and dried soil (Ferraro & Ghersa, 2007).

We determined soil enzymatic activity using the methods described by Guan (1986). Urease activity was measured using a pH 6.7 citrate acid buffer solution at 37 °C for 24 h. Catalase activity was determined by shaking soil samples with potassium permanganate titration at 25 °C for 20 min. We determined acid-phosphatase activity using P-nitrophenyl phosphate disodium at 37 °C for 24 h. Invertase activity was determined using 3, 5-dinitro-salicylic acid colorimetry at 37 °C for 24 h. We measured soil microbial quantity using the methods described by Xu & Zheng (1986). First, 10 grams of fresh soil material were shaken well with 90 mL of sterilized saline solution (8.50 g L^−1^ NaCl), and serial dilutions (10^−1^ to 10^−5^) were made in sterile saline solution. Then, we counted the number of bacteria, actinomyces, and fungi using nutrient agar, starch nitrate medium, and potato dextrose agar, respectively. Bacteria were cultured at 37 °C for 2 days, and actinomyces and fungi were cultured under aerobic conditions at 37 °C for 3 days.

### Statistical analysis

We used repeated measures ANOVA to test the effects of planting year, sowing methods, crop treatments, and their interactions on soil properties and crop yields (***, significant at *P* < 0.001; **, significant at *P* < 0.01; *, significant at *P* < 0.05; NS, not significant). The means were then compared for significance using Duncan’s multiple range method. All statistical procedures were conducted using the statistical packages SPSS (SPSS 17.0 for Windows, SPSS Inc., Chicago, IL, USA).

## Results

### Soil characteristics and enzymatic activity

The soil pH, bulk density, organic matter, and enzymatic activity were different across the experiment years (*P* < 0.05), but crop type had no effect on these soil factors (*P* > 0.05) (Table 3). NB5’s soil pH was lower than that of CK (*P* < 0.05), while its soil bulk density was higher than that of CK (*P* < 0.05). NA1’s soil organic matter and total nitrogen content were higher than those of CK (*P* < 0.05). We found no significant total nitrogen content differences across experiment years. The soil urease, catalase, and invertase activity in 2016–2017 was lower than in 2017–2018 (*P* < 0.05). Although urease and catalase activity was not statistically different across the sowing methods, acid-phosphatase and invertase activity in both NB5 and NA1 was lower than in CK (*P* < 0.05). NB5 and NA1 had similar soil total nitrogen content and enzyme activity. Bacteria and actinomyces were not significantly affected by experiment year and sowing methods (*P* > 0.05) (Table 4). Generally, the number of microorganisms in the conventional tillage soil was higher than in the no-tillage soil.

**Table 3:** pH, bulk density, organic matter, total nitrogen and enzyme activities for different treatments and crop types.

**Year and treatments**		**pH**	**Bulk density (g cm^−3^)**	**Organic matter (g kg^−1^)**	**Total nitrogen (g kg^−1^)**	**Urease (mg g^−1^24 h^−1^)**	**Catalase (mg g^−1^ 20 min^−1^)**	**Acid–phosphatase (mg g^−1^24 h^−1^)**	**Invertase (mg g^−1^ 24 h^−1^)**
Year	2016–2017	5.34 ± 0.04a	1.38 ± 0.02a	24.53 ± 0.32a	1.44 ± 0.06	0.33 ± 0.01b	0.94 ± 0.05b	72.45 ± 1.86a	7.32 ± 0.35b
	2017–2018	5.14 ± 0.02b	1.24 ± 0.03b	22.73 ± 0.37b	1.32 ± 0.05	0.55 ± 0.02a	2.57 ± 0.06a	57.05 ± 1.48b	11.17 ± 0.71a
Sowing method	NB5	5.15 ± 0.04b	1.32 ± 0.03ab	23.42 ± 0.43b	1.43 ± 0.07a	0.44 ± 0.03	1.60 ± 0.22	62.16 ± 2.86b	8.19 ± 0.55b
	NA1	5.27 ± 0.05ab	1.37 ± 0.04a	24.92 ± 0.46a	1.51 ± 0.05a	0.40 ± 0.03	1.80 ± 0.28	61.22 ± 2.99b	8.47 ± 0.69b
	CK	5.31 ± 0.06a	1.25 ± 0.03b	22.56 ± 0.34b	1.19 ± 0.05b	0.47 ± 0.05	1.85 ± 0.25	70.87 ± 2.63a	11.07 ± 1.11a
Crop	Forage wheat	5.27 ± 0.05	1.34 ± 0.02	23.87 ± 0.41	1.39 ± 0.06	0.46 ± 0.03	1.82 ± 0.20	66.89 ± 2.85	8.93 ± 0.77
	Italian ryegrass	5.22 ± 0.04	1.28 ± 0.03	23.40 ± 0.39	1.37 ± 0.05	0.42 ± 0.03	1.68 ± 0.21	62.61 ± 1.98	9.56 ± 0.68
*P* value	Year (Y)	***	***	**	NS	***	***	***	***
	Sowing method (SM)	*	*	**	**	NS	NS	*	*
	Crop (C)	NS	NS	NS	NS	NS	NS	NS	NS
	Y × SM	*	*	**	**	**	NS	**	***
	Y × C	NS	**	NS	NS	*	NS	NS	NS
	SM × C	***	***	***	NS	***	***	***	***
	Y × SM × C	**	**	NS	**	**	***	***	**

**Notes.**

Different lowercase letters in the same column represent significant difference between experiment years, sowing methods or crop treatments (*P* < 0.05),*** , significant at *P* < 0.001; **, significant at *P* < 0.01;*, significant at *P* < 0.05.

NS not significant NB5 notillage sowing on the 5 d before rice harvest NA1 notillage sowing on the 1d after rice harvest CK conventional tillage sowing on the 1d after rice harvest

**Table 4:** Measurements of soil microorganisms for different treatments and crop types.

**Year and treatments**		**Bacteria (lg cfu g^−1^ FM)**	**Actinomyces (lg cfu g^−1^ FM)**	**Fungi (lg cfu g^−1^ FM)**
Year	2016–2017	7.41 ± 0.22	6.08 ± 0.14	4.99 ± 0.08
	2017–2018	6.85 ± 0.18	5.88 ± 0.07	4.96 ± 0.06
Sowing method	NB5	6.95 ± 0.27	5.87 ± 0.11	4.83 ± 0.56b
	NA1	6.88 ± 0.23	5.83 ± 0.07	4.83 ± 0.08b
	CK	7.56 ± 0.24	6.24 ± 0.18	5.27 ± 0.06a
Crop	Forage wheat	7.81 ± 0.17a	6.29 ± 0.11b	4.97 ± 0.07
	Italian ryegrass	6.46 ± 0.08b	6.67 ± 0.45a	4.98 ± 0.08
*P* value	Year (Y)	NS	NS	NS
	Sowing method (SM)	NS	NS	***
	Crop (C)	***	***	NS
	Y × SM	NS	NS	***
	Y × C	***	***	NS
	SM × C	NS	NS	NS
	Y × SM × C	**	*	NS

**Notes.**

Different lowercase letters in the same column represent significant difference between experiment years, sowing methods or crop treatments (*P* < 0.05),***, significant at *P* < 0.001; **, significant at *P* < 0.01; *, significant at *P* < 0.05.

NS not significant NB5 notillage sowing on the 5 d before rice harvest NA1 notillage sowing on the 1d after rice harvest CK conventional tillage sowing on the 1d after rice harvest FM fresh matter

### Soil capillary porosity and water content

**Figure 2:**
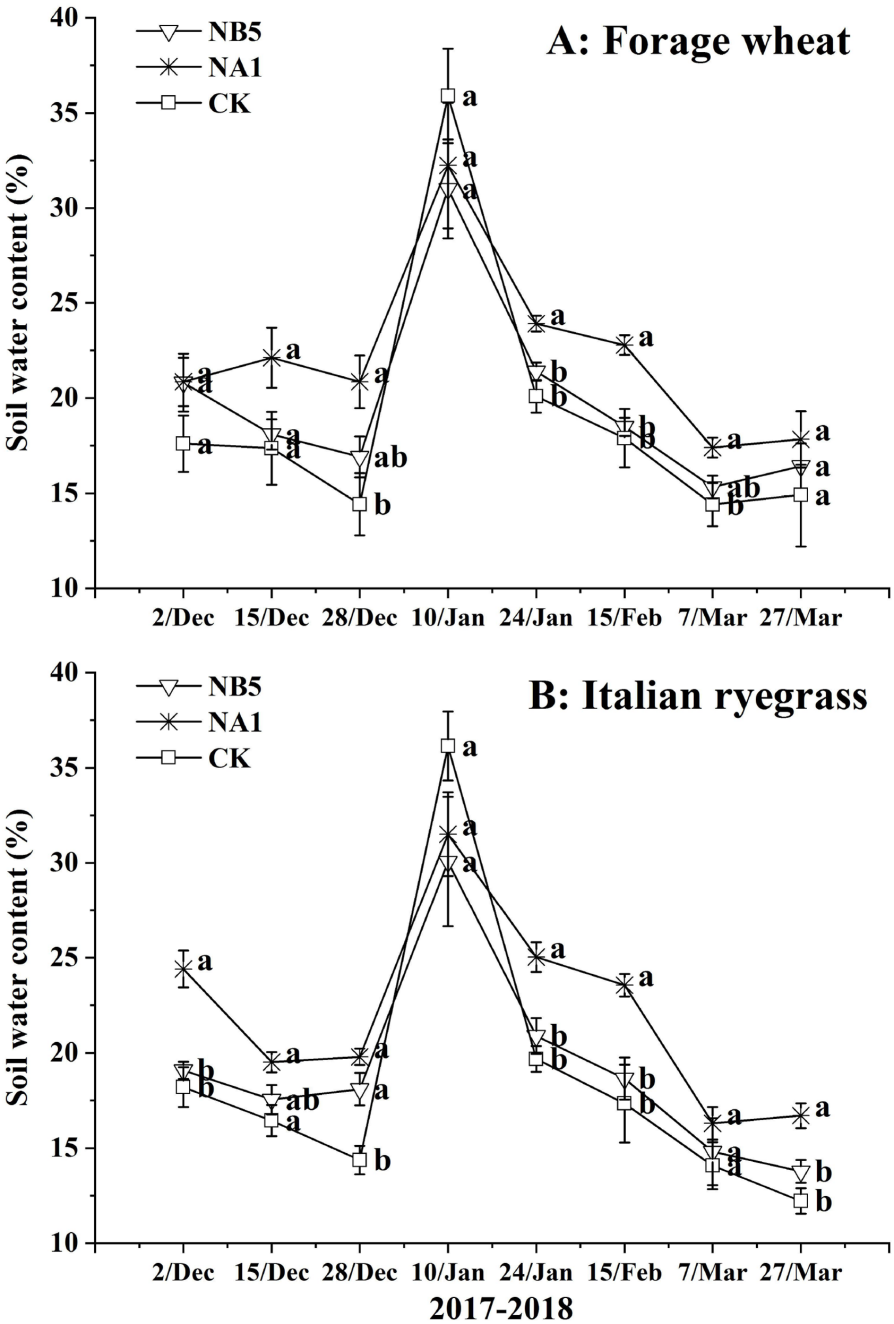
Changes of soil water content during wheat (A) and Italian ryegrass (B) growing period during 2017–2018. Different lowercase letters associated with the same time indicate the difference of significance in the level of *P* < 0.05, and the error bars denote the standard error of data. NB5: no–tillage sowing on the 5 d before rice harvest; NA1: no–tillage sowing on the 1d after rice harvest; CK: conventional tillage sowing on the 1d after rice harvest.

We observed no differences in capillary moisture capacity and non-capillary soil porosity across experiment years and between sowing methods (*P* > 0.05). However, we did find differences for these two factors in the interaction between experiment year and sowing methods (*P* < 0.05). During the whole growing period, NB5 and NA1 had greater soil water storage, field moisture capacity, relative water content, and capillary porosity than CK (*P* < 0.05) (Table 5). The forage wheat and Italian ryegrass treatments had similar soil physical properties, excluding capillary moisture capacity. We observed the highest water content in NA1, followed by NB5 and CK in both forage wheat and Italian ryegrass fields (Fig. 2).

**Table 5:** Measurements of soil physical properties for different treatments and crop types in 2017–2018.

**Time and treatments**		**Bulk density (g cm^−3^)**	**Soil water storage (mm)**	**Field moisture capacity (%)**	**Relative water content (%)**	**Total porosity (%)**	**Capillary moisture capacity(%)**	**Capillary porosity(%)**	**Non–capillary porosity (%)**
Time	Jan 24	1.18 ± 0.03	148.49 ± 6.92a	34.90 ± 1.87a	70.47 ± 1.92a	55.52 ± 1.02	37.25 ± 1.11	44.29 ± 0.96	9.02 ± 1.17
	Feb 25	1.23 ± 0.03	123.17 ± 6.00b	29.50 ± 1.00c	65.57 ± 2.48a	53.24 ± 1.13	35.50 ± 1.24	43.78 ± 0.68	9.63 ± 1.10
	Mar 27	1.24 ± 0.02	97.58 ± 6.00c	30.22 ± 0.92b	47.71 ± 2.12b	53.29 ± 0.89	35.90 ± 0.98	44.14 ± 0.76	8.68 ± 0.81
Sowing method	NB5	1.24 ± 0.03a	128.21 ± 6.51b	32.84 ± 0.89a	61.24 ± 2.36b	52.87 ± 0.73b	36.59 ± 0.93	44.66 ± 0.72a	8.20 ± 0.66
	NA1	1.28 ± 0.02a	147.78 ± 5.61a	34.26 ± 1.92a	69.43 ± 3.21a	51.93 ± 1.13b	36.97 ± 1.44	45.72 ± 0.86a	9.27 ± 1.08
	CK	1.13 ± 0.02b	93.25 ± 5.07c	27.51 ± 0.63b	53.09 ± 2.74c	57.24 ± 0.74a	35.09 ± 0.87	41.83 ± 0.54b	9.84 ± 1.26
Crop	Forage wheat	1.25 ± 0.02	130.19 ± 5.86	32.42 ± 1.45	64.61 ± 2.25	53.57 ± 0.93	34.95 ± 0.85b	44.29 ± 0.59	8.48 ± 0.80
	Italian ryegrass	1.19 ± 0.02	115.97 ± 7.04	30.66 ± 0.77	57.89 ± 2.77	54.46 ± 0.75	37.48 ± 0.91a	43.85 ± 0.71	9.73 ± 0.87
*P* value	Time (T)	NS	***	*	***	NS	NS	NS	NS
	Sowing method (SM)	***	***	**	**	***	NS	**	NS
	Crop (C)	NS	NS	NS	NS	NS	*	NS	NS
	T × SM	***	***	***	***	***	NS	**	NS
	T × C	NS	NS	*	NS	NS	NS	NS	NS
	SM × C	NS	NS	NS	NS	NS	***	*	***
	T × SM × C	NS	**	***	**	NS	NS	NS	*

**Notes.**

Different lowercase letters in the same column represent significant difference between experiment years, sowing methods or crop treatments (*P* < 0.05),***, significant at *P* < 0.001; **, significant at *P* < 0.01;*, significant at *P* < 0.05.

NS not significant NB5 notillage sowing on the 5 d before rice harvest NA1 notillage sowing on the 1d after rice harvest CK conventional tillage sowing on the 1d after rice harvest

### Dry matter and crude protein yield

Although there were no differences found for DM yield, tiller number per plant, and plant heights across the experiment years and between sowing methods (*P* > 0.05), we did find a significant difference in the crop population density (Table 6). Compared to Italian ryegrass, forage wheat had a higher DM yield, population density, and plant height (*P* < 0.05). The interaction between experiment year and crop type was significant for the forage wheat DM and CP yield. However, the experiment year and sowing method had no effect on DM yield, plant height, or tiller number per plant (*P* > 0.05). Crops in 2017–2018 had lower CP yield and population density than crops in 2016–2017 (*P* < 0.05). Compared to NB5, NA1 and CK had slightly higher DM and CP yields. The population density of both NB5 forage wheat and Italian ryegrass was higher than that of NA1 and CK (*P* < 0.05).

**Table 6:** Plant height, tiller number, population density and yield for different treatments and crop types.

**Year and treatments**		**Dry matter yield (t ha^−1^)**	**Crude protein yield (t ha^−1^)**	**Population density (10^6^ plant ha^−1^)**	**Plant height (cm)**	**Tiller number per plant**
Year	2016–2017	8.13 ± 0.68	0.99 ± 0.46a	2.81 ± 0.39a	67.23 ± 4.51	6.93 ± 1.65
	2017–2018	8.32 ± 0.21	0.74 ± 0.40b	1.84 ± 0.21b	70.19 ± 3.03	7.51 ± 1.60
Sowing method	NB5	7.53 ± 0.86	0.82 ± 0.05	3.23 ± 0.53a	67.34 ± 5.30	4.57 ± 0.96
	NA1	8.72 ± 0.39	0.84 ± 0.09	1.63 ± 0.18b	68.78 ± 4.44	8.81 ± 2.49
	CK	8.43 ± 0.47	0.93 ± 0.04	2.11 ± 0.28b	70.01 ± 4.58	8.29 ± 2.04
Crop	Forage wheat	9.55 ± 0.32a	0.93 ± 0.06	3.18 ± 0.37a	83.58 ± 0.96a	1.48 ± 0.05b
	Italian ryegrass	6.90 ± 0.45b	0.80 ± 0.02	1.47 ± 0.05b	53.84 ± 1.69b	12.96 ± 1.18a
*P* value	Year (Y)	NS	***	*	NS	NS
	Sowing method (SM)	NS	NS	*	NS	NS
	Crop (C)	***	NS	***	***	***
	Y × SM	NS	*	NS	NS	NS
	Y × C	***	**	*	**	NS
	SM × C	*	NS	**	NS	***
	Y × SM × C	NS	***	***	NS	NS

**Notes.**

Different lowercase letters in the same column represent significant difference between experiment years, sowing methods or crop treatments (*P* < 0.05),***, significant at *P* < 0.001; **, significant at *P* < 0.01; *, significant at *P* < 0.05.

NS not significant NB5 notillage sowing on the 5 d before rice harvest NA1 notillage sowing on the 1d after rice harvest CK conventional tillage sowing on the 1d after rice harvest

## Discussion

### Soil characteristics and enzymatic activities

In this study, the soil pH of the no-tillage field was slightly higher than the conventionally tilled field. Generally, leaves that came into contact with the soil surface or that had been decomposed into the soil can absorb Al^3+^ and H^+^ and regulate soil pH (Garbuio et al., 2011). Pocknee & Sumner (1997) found that adding plant residues to soil can improve cation exchange capacities because the residues are likely to absorb significant amounts of Al^3+^ and H^+^ from the mineral soil and therefore partially contribute to the neutralization effect. Although the decomposition rate of mulch on the surface of the no-tillage soil was lower than that of the tillage soil, the combination of mulch with the surface layer of the soil may have had a greater impact on Al^3+^ and H^+^.

Long-term no-tillage sowing increased the organic carbon content of the soil surface compared to conventional tillage (Chen et al., 2009). In this study, we found that short-term no-tillage sowing also increased the soil organic matter and total nitrogen content in NA1 and NB5. Despite the same quantity of rice straw being returned to the field under both no-tillage and conventional treatments, the no-tillage cultivation may have protected the soil structure and suppressed the decomposition rate of soil organic matter (Mccarty, Lyssenko & Starr, 1998; Hou et al., 2012). This result was similar to the findings of Larney & Bullock (1994). It has been shown that an increase in experimental time accelerates the carbon sequestration rate in no-tillage fields in South China, and the accumulation of soil organic carbon was significantly higher than that of the conventionally tilled fields (Chen, 2017). No-tillage sowing inhibits microbial decomposition by reducing soil disturbance, which also increases soil organic matter content (Chatskikh & Olesen, 2007; Chen et al., 2009). On the other hand, NB5 and NA1 had higher soil bulk densities than CK (*P* < 0.05) in this study. The no-tillage soil had a higher bulk density, which resulted in the plant roots being mainly distributed in shallow soil (Martínez et al., 2008). Conventional tillage caused a large amount of organic matter to be exposed on the surface, which accelerated the rate of mineralization and decomposition (Franzluebbers & Arshad, 1996; Islam & Weil, 2000). Although we did not assess the mineralization and decomposition of soil organic matter in this study, we did not exclude these factors when evaluating the decrease of organic matter content using conventional tillage.

Enzyme activity can be used as an important indicator of soil nutrient cycling and energy transfer (Mdela et al., 2002). The availability of soil enzymes and nutrients are regulated and controlled by soil microbes (Figuerola et al., 2012). Soil’s ability to supply crop nutrients depends on the availability of potential nutrients, which is related to soil enzyme activity. Soil urease is an important hydrolase for converting organic nitrogen to effective nitrogen, which is critical for improving the nitrogen utilization rate and promoting the soil nitrogen cycle. Invertase can also be used as an indicator of soil fertility level. Phosphatase accelerates the dephosphorization rate of soil organic phosphorus. Some studies have reported that no-tillage sowing has a positive effect on soil enzyme activity (Ekenler & Tabatabai, 2003; Zuber & Villamil, 2016). However, Chatskikh & Olesen (2007) found that no-tillage sowing inhibited the activity of soil microbes. Our results showed that no-tillage sowing did not increase enzyme activity in forage wheat or Italian ryegrass, (Table 3), indicating that no-tillage sowing reduced microbial activity (Table 4) but improved soil water storage, field moisture capacity, relative water content, and capillary porosity (Table 5).

### Soil capillary porosity and water content

Previous studies have found that long-term, no-tillage sowing improved soil quality by enhancing soil structure, increasing water supply capacity, and reducing soil erosion (He et al., 2011; Mikha, Vigil & Benjamin, 2013), while short-term, no-tillage sowing reduced soil disturbance and decreased water evaporation from the soil in semi-arid areas (Yang et al., 2018) and winter fallow paddy fields (Chen et al., 2008). Our results showed that no-tillage fields had higher soil water storage, field moisture capacity, and relative water content than conventionally tilled fields (Table 4). This may be partly because of the gas permeability caused by no-tillage sowing (Debaeke & Aboudrare, 2004; He et al., 2011). We observed the highest water content in the NA1-treated soil (Fig. 2), perhaps due to the fact that no-tillage sowing and low crop population density decreased soil water evaporation and plant transpiration. The sowing date can also affect the water distribution pattern during crop growth. In this study, we found that early sowing increased the crop population density and aboveground biomass. This strengthened the plant transpiration, which decreased the available soil water content of crops at the maturity stage. CK crops generally had a higher population density than the NA1 crop. Also, CK evaporated more soil water through soil disturbance, so the soil water content of the CK crops was mostly lower than that of the NB5 and NA1 crops. Additionally, rainfall did influence all of the treatments. CK’s soil water content was higher than that of NB5 and NA1 (Fig. 2) and correlated with each month’s rainfall period (Fig. 1). For example, rainfall was high in January 2018 and because CK had a higher soil water absorption rate than the no-tillage fields, CK had higher soil water content during this period.

### Forage yield

Xu et al. (2008) found that early sowing (5–14 days before harvest) increased the population density of forage wheat in paddy fields. Although NA1 had the lowest population density in our study, NA1 may still be beneficial for the accumulation of photosynthetic products in individual plants (Tollenaar et al., 1994) and offsetting declines in yield due to low population density. The CP yield of forage wheat crops in 2017–2018 was lower than that of crops in 2016–2017 (*P* < 0.05), due to less rainfall (137.7 mm vs. 202.7 mm) and fewer rainy days (17 days vs. 31 days) during key growth periods (seedling and filling stages). NB5 had the lowest DM and CP yields. An increase in wheat population density can also increase the competition for water, light, and nutrients, and can change the stomatal conductance, intercellular carbon dioxide concentration, and chlorophyll content of wheat leaves, which can eventually lead to the accumulation of assimilates (Moreira et al., 2015).

It is worth noting that no-tillage sowing also increased the number of weed species (*Polygonum aviculare, Stellaria media, Alopecurus aequalis, Chenopodium glaucum, Alternanthera philoxeroides,* and *Centipeda minima*) and aboveground biomass (Table 7), which also affect forage yield. We cultivated Italian ryegrass as a green manure or forage crop in the winter fallow paddy fields, although the conditions were extremely competitive and the plants hardly survived when the paddy fields were flooded during subsequent rice cultivation.

**Table 7:** Species diversity and weed dry matter yield in 2016–2017 for different treatments and crop types.

**Sowing method and crops**		**Shannon’s diversity index**	**Margalef index**	**Simpson index**	**Pielou index**	**Dry matter yield (g m^−2^)**
Sowing method	NB5	1.34 ± 0.03b	1.89 ± 0.02b	0.23 ± 0.01b	1.16 ± 0.19	7.82 ± 0.59b
	NA1	1.57 ± 0.02a	3.30 ± 0.14a	0.29 ± 0.01a	1.33 ± 0.24	10.66 ± 0.67a
	CK	1.11 ± 0.07b	1.71 ± 0.30b	0.19 ± 0.01c	0.79 ± 0.05	4.76 ± 0.24c
Crop	Forage wheat	1.41 ± 0.06	2.34 ± 0.27	0.22 ± 0.19	1.44 ± 0.15a	8.66 ± 1.03
	Italian ryegrass	1.27 ± 0.08	2.26 ± 0.25	0.25 ± 0.02	0.75 ± 0.03b	6.84 ± 0.74
*P* value	Sowing method (SM)	***	***	***	NS	***
	Crop (C)	NS	NS	NS	**	NS
	SM × C	**	NS	NS	***	NS

**Notes.**

Different lowercase letters in the same column represent significant difference between experiment years, sowing methods or crop treatments (*P* < 0.05),***, significant at *P* < 0.001;**, significant at *P* < 0.01;*, significant at *P* < 0.05.

NS not significant NB5 no-tillage sowing on the 5 d before rice harvest NA1 no-tillage sowing on the 1d after rice harvest CK conventional tillage sowing on the 1d after rice harvest

When plants were only cut once, forage wheat tended to have higher DM and CP yields than that of the Italian ryegrass. This may be because of forage wheat’s higher plant height and population density. Despite forage wheat’s relatively higher DM and CP yield, Italian ryegrass had the advantage when plants were harvested or grazed multiple times. NA1’s population density was lower than that of CK. However, there were no differences in DM and CP yields between NA1 and CK (*P* > 0.05), indicating that NA1 had a greater DM accumulation rate per plant than CK. Wheat had a lower water use during the vegetative growth stage, and water stored in soil triggers the accumulation of higher aboveground biomass during the reproductive growth stage. Other studies on no-tillage sowing found that sufficient soil water content had a significant impact on grain yield (cereal crops) (Zhang et al., 2019) and aboveground biomass (forage) (Zhang et al., 2017), and the accumulation of these yields or aboveground biomass significantly and positively correlated with the crop’s water use efficiency.

## Conclusion

The results of this study show that planting forage wheat and Italian ryegrass after harvesting rice generally improved soil nutrient status when compared to winter fallowing. Conventional tillage tended to increase soil enzyme activity and no-tillage sowing increased soil water, organic matter, and total nitrogen content. No-tillage sowing resulted in slightly lower DM and CP yields than conventional tillage, though the difference was not significant. No-tillage sowing led to greater species richness and aboveground weed biomass compared to conventional tillage. Forage wheat had a higher DM yield than Italian ryegrass under the same conditions. The best sowing method for forage wheat and Italian ryegrass in winter fallow paddy fields was no-tillage sowing following the rice harvest. The results of this study provide useful information for rice growing and cropping management alternatives to fallowing for forage wheat and Italian ryegrass.

##  Supplemental Information

10.7717/peerj.10573/supp-1 Supplemental Information 1 Plant and soil data

## Additional Information and Declarations

### Data Availability

The following information was supplied regarding data availability: Raw measurements are available in the Supplemental File.
